# Is the Annual Confidential Report system effective? A study of the government appraisal system in Gujarat, India

**DOI:** 10.1186/s12960-016-0133-8

**Published:** 2016-06-02

**Authors:** Bhaskar Purohit, Tim Martineau

**Affiliations:** Indian Institute of Public Health Gandhinagar (IIPHG), Sardar Patel Institute Campus, Drive in Road, Thaltej, Ahmedabad, 380054 India; Liverpool School of Tropical Medicine (LSTM), Pembroke Place, Liverpool, L3 5QA United Kingdom

## Abstract

**Background:**

Effective performance appraisal systems can not only motivate employees to improve performance but also be important for the performance of organizations. However, the appraisal systems in civil services called the Annual Confidential Report (ACR) systems can be ineffective and do not contribute to employees’ learning and development. With this background, the current study aimed at understanding the ACR system and assessing its effectiveness. The research aims to contribute in filling the knowledge gap in the existing literature on the need as to why the ACR system in civil services is an important human resource management (HRM) function.

**Methods:**

The analysis is based on policy review to understand the extant appraisal-related rules and policies. Nineteen in-depth interviews with medical officers (MOs) working with the government health department of Gujarat, India, were conducted. The main objective of the research was to assess the effectiveness of the actual appraisal system called or referred to as the ACR as perceived by MOs. Thematic framework approach was used to analyze qualitative data using NVIVO 9. Themes were built around five features of an effective appraisal system, i.e., purpose, source, feedback quality, link of the ACR system with other human resource functions, and administrative effectiveness.

**Results:**

The five features of the effective appraisal system studied in the current research (purpose, source, feedback quality, link of ACR system with other HRM functions, and administrative effectiveness) indicate that the overall appraisal system is ineffective. The overall appraisal system was perceived to be subjective and one directional in character by the study respondents. Furthermore, respondents perceived the appraisal system to be a ritual and where MOs hardly got to know about their performance, especially good performance. Hence, the feedback loop, an important feature for an effective appraisal system, was absent. The overall ACR system functions in isolation with no link to other HRM functions such as training and counselling, and a weak link with salary administration and promotion.

**Conclusions:**

Addressing the five features or domains of an effective appraisal system can lead to improved perceived fairness MOs have on the current appraisal system which may further influence the satisfaction and motivation positively. Improved motivation and satisfaction with the appraisal system can influence two important human resource for health-related outcomes, i.e., performance and retention.

## Background

Although issues surround the health workforce, mostly concerning increasing the numbers, lateral and equitable distribution have received increased attention in the last decade. However, to complement these improvements, it is also necessary to focus on health worker performance.

There are several ways of improving staff performance, one of which is performance appraisal (PA). Performance appraisal takes numerous forms, but in general and in a simple way, performance appraisal may be defined as the process through which the performance of employees is measured and improved [1]. Since the focus of performance appraisal is not only on the assessment of performance but also on the improvement of performance, performance appraisal includes various practices like the recognition of employees’ achievements and providing them feedback for personal and professional development [2]. Literature on performance appraisal suggests that a good performance appraisal has mainly the following objectives and functions: (1) administrative functions which are concerned with taking decisions regarding salary administration, promotion, and rewards [1, 3]; (2) formal assessment of performance in order to suggest improvements for employee productivity [1, 4]; (3) development of an employee’s competencies and capabilities through training, learning, and career planning [1, 5]; and (4) overall job analysis so that individual performance can be linked to the development needs of the job [1].

**Fig. 1:**
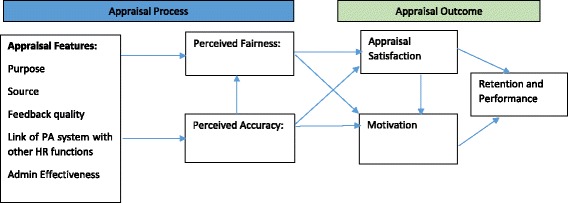
Conceptual framework for the study

Literature from management suggests that performance appraisal is an important human resource management (HRM) function [1, 6, 7] and is important for organizational effectiveness [1, 8]. Effective performance appraisal systems have been identified as important for the performance of organizations, and ineffective appraisal systems have been associated with reduced employee morale and decreased employee productivity [9]. Therefore, it becomes important to assess the effectiveness of appraisal systems. Several criteria may be used to assess the effectiveness of a performance appraisal system such as (1) the perceived accuracy of appraisals by employees, (2) the employees’ perception of fairness with the appraisal system [10, 11], (3) the appraisal source which suggests employee evaluation of performance through various sources involved in the appraisal process [12], (4) the purpose of the appraisal system [13], and (5) the feedback richness in the appraisal [14]. All these criteria are presented in the conceptual framework of the study in Fig. 1. However, the effectiveness of the performance appraisal will depend not only on the abovementioned criteria and design of the system but also the way in which the appraisal functions are carried out. There could be several types of appraisal systems as explained below:

### Annual Confidential Reports

The Annual Confidential Report (ACR) system is an old system started in the 1940s but still used in the public sector organizations of many middle- and low-income countries (MLICs) such as India, Swaziland, and Sri Lanka [15]. It is done annually and mainly carried out for promotion-related decisions where promotions are based on seniority subject to satisfactory ACR. ACR reports are confidential, and the employees do not generally get an opportunity to discuss their performance with their seniors [15].

### Management by objectives

Management by objective (MbO) is primarily concerned about the achievement of objectives and allows the managers to know what is being expected from them. But the MbO focuses on results and fails to notice the job behavior [16].

### Performance appraisal

Due to problems in the MbOs, performance appraisal systems were introduced in the 1970s. The performance appraisal system seeks to improve the problems and procedures of ACR and MbOs by mainly improving the quality of data for appraisal decisions with the opportunity for feedback to the employees [15].

### 360-degree appraisal

In the 360-degree appraisal system, information is obtained through several sources which includes the boss, top management, assistants, coworkers, customers, community, etc. 360-degree appraisal systems can be more effective as compared to the previous systems that were one sided and could be biased at times [1].

In India, the government employs majority of the health workers. Across India, the government relies on a common appraisal system which is called the ACR systems which is used to assess the performance of all the government employees from different departments. As ACR is largely the only government system for managing staff performance, it is pertinent to ask the following questions: (1) whether the design of ACR is appropriate or not and (2) how effective is the ACR system. Therefore, the current study aimed at understanding the current appraisal system of ACR used for Medical Officers (MOs) from the health department of the Gujarat state in India and assess its effectiveness as perceived by MOs by using the conceptual framework presented in Fig. 1.

### Conceptual framework

Performance appraisal systems can have a great impact on decisions related to the salary, career prospects, and development of employees and can have important consequences for the motivation of employees which can in turn influence the performance of employees. The theoretical model for this research is presented in Fig. 1. The current study aimed at assessing the overall effectiveness of the appraisal system known as the ACR system followed in civil services in India for doctors employed with the government health department in Gujarat, India. To assess overall effectiveness, the study aimed at studying several appraisal characteristics such as the source of appraisal, the overall purpose and usefulness of the appraisal systems, and the feedback quality [17]. A framework developed by Selvarajan and Peggy was adapted for the study [17].

The three appraisal characteristics, i.e., source, purpose, and feedback, have been found to be important variables in influencing the perceptions about fairness and accuracy employees have about the appraisal system and are critical features of appraisal systems in assessing their effectiveness [17]. The appraisal source in the framework suggests employee evaluation of performance through various sources involved in the appraisal process [12]. The purpose of the appraisal system suggests and questions the main objective of the appraisal system [13]. The feedback richness in the appraisal means a system where employees receive specific, frequent, and timely feedback [14].

In order to assess effectiveness, two additional appraisal characteristics were added to the adapted framework: (1) link of appraisal system with other HRM functions and (2) administrative effectiveness of the actual process followed in the appraisal system. The link of appraisal system with other HRM functions has been identified as very critical for organizational effectiveness [1]. Also, administrative effectiveness suggests the way in which the appraisal functions are carried out and can have influence over appraisal outcomes such as satisfaction, motivation, and intentions to quit or continue in a job.

The conceptual framework presents all the five characteristics of effectiveness (purpose, source, feedback quality, link of ACR system with other human resource functions, and administrative effectiveness) as they mainly relate to the process of the appraisal system. These appraisal characteristics affect the perceived accuracy and perceived fairness employees have on the appraisal system. The perceived accuracy of appraisals by employees is one of the main criteria used to assess the effectiveness of the appraisal system [18] and is linked to various appraisal outcomes such as the employee’s satisfaction with the appraisal system and their motivation to improve performance [19, 20]. Perceived accuracy means how the employees perceive the appraisal system to be accurate in measuring what it is intended to measure and whether the appraisal system meets its objectives.

Employees’ perceptions about the accuracy of the appraisal system (called perceived accuracy) influence their perceptions about the fairness of the appraisal system (called perceived fairness). The perceived accuracy and perceived fairness of the employees also influence two main outcomes of an appraisal system. At the outcome level, additional Human Resource for Health (HRH) outcomes, i.e., performance and retention, were added to the adapted model as satisfaction with the appraisal system as well as motivation can lead to better retention and performance.

Although the current study did not directly assess the appraisal process relating to perceived accuracy, and the appraisal outcomes, nonetheless, the responses from the study respondents suggest how they perceive the current appraisal systems in light of their usefulness and in meeting the desired objectives of the appraisal which indicate a potential link of the appraisal-related processes with the appraisal outcomes.

Literature suggests that the appraisal systems or the ACR in civil services has been found to be ineffective and does not contribute to employees’ learning and development as the ACR system has communication gaps with personal biasness and lack of employees’ participation [16]. With this background, the current study aimed at understanding the current appraisal system or the ACR and assessing its effectiveness as perceived by the MOs in the state of Gujarat, India, by using the above conceptual framework.

Gujarat state was selected for this study as it represents the economically progressive states of India with health indicators much better than the national average, yet the state suffers from shortage of MOs and specialists, especially in rural areas [21]. The vacancy and shortfall in the state is 24 % for MOs at Primary Health Centers (PHCs) while the vacancy and shortfall is particularly high (77 % and 93 %, respectively) for all specialists working with Community Health Centers (CHCs) [21] Another reason for selecting Gujarat was the presence of a public health institute in the state called the Indian Institute of Public Health Gandhinagar (IIPHG) that is working closely with the Department of Health, Government of Gujarat, to strengthen the health system in the state. The selection of Gujarat not only served the mandate of IIPHG to strengthen the health system in the state through research but seeking permission for the study from the state was also easier.

### The health system in Gujarat

As per the state’s Civil Services Recruitment Rules 1967, the MOs have been categorized into two classes: I and II. Both classes I and II are gazetted posts, and the state’s Public Service Commission (PSC) called the Gujarat Public Service Commission (GPSC) is responsible for the recruitment of all gazetted posts including MOs [22]. All the graduate doctors are recruited as MOs in class II whereas those holding postgraduate degree in clinical areas are recruited as specialist class I. All specialist and senior positions at the district and state levels are class I positions while the MOs working with PHCs and CHCs without postgraduate specialization are class II positions. According to health service norms, each CHC needs to be staffed with specialists as well as regular doctors or MOs. Similarly, each PHC needs to be staffed with at least one MO.

At the district level, the Chief District Health Officer (CDHO) who is a class I officer is overall in charge of the CHCs and PHCs within the district. *Several blocks or the administrative units constitute a district*. Blocks are administered by the Block Health Officers (BHOs) which is also a class II position.

#### Bonded medical officers

Under the compulsory rural service in Gujarat, all the medical graduates from the government colleges enter the government service under the “bonded” category and are required to sign a bond at the time of admission to medical college that requires them to compulsorily serve in rural areas for 2 years.

#### Ad hoc medical officers

To address the shortage of MOs in the state, the Department of Health and Family Welfare in the past recruited MOs from Gujarat such as candidates from private medical colleges or outside the state. Recruitment of such MOs is called ad hoc appointment. MOs under ad hoc appointment were appointed on a temporary basis and are required to pass the states’ PSC exam called GPSC in order to be appointed as permanent employees which would give them regular service.

#### Contractual medical officers

Yet another category under which MOs are recruited is called the “contractual appointment” where the appointments are done for 11 months. The contractual category includes all the MOs from private medical colleges or from outside the state or the ones who do not meet the age criterion of the GPSC but who wish to work with the government health department. Contractual MOs may also include MOs from alternative systems such as homeopathy or Ayurveda. The MOs under the contractual category do not get employment benefits which are otherwise available to MOs who are on “regular service” till the time they pass the states’ PSC exam.

#### District hospital

The district hospital (DH) is a public hospital that caters to the health needs of the entire district providing mainly tertiary care.

#### Community health center

A CHC is a 30-bed hospital that constitutes the secondary level of health care and provides referral as well as specialist health care to the rural population at the block level. It caters to 80 000–120 000 population.

#### Primary health center

A PHC covers a population of 20 000 in hilly, tribal, or difficult areas and 30 000 populations in plain areas with four to six indoor/observation beds. It acts as a referral unit for six sub-centers and refers out cases to the CHC (30-bed hospital) and higher order public hospitals located at the sub-district and district levels.

## Methods

### Study design

The current research was part of a larger research study to assess various HRM systems, policies, and practices such as recruitment, placement, posting and transfer, and appraisal where qualitative methods of research were used. For the purpose of the current study on appraisal, policy review was carried out to understand the ACR rules. Nineteen in-depth interviews with MOs were conducted to assess ACR systems (processes and practices) and MOs’ perceptions about the ACR system and to assess the effectiveness of the ACR system based on five features of the effective appraisal system. In-depth qualitative methods were best suited to the scope of current study as the study aimed at organizing the data into themes presented in the “Results” section [23].

### Study setting

This study was conducted in the state of Gujarat, India, in 2013. MOs were included as the study subjects working for the government health department placed at rural health centers from three different districts from the state selected based on purposive sampling. Based on initial discussions with several MOs and state-level officers prior to the study, a list of a few “desirable,” “not so desirable,” and “not at all desirable” districts for MO posting was made. The main criteria for “desirable,” “not so desirable,” and “not at all desirable” districts were mainly doctors’ willingness in general to be posted to such districts. The doctors’ willingness to be posted is influenced by factors such as close proximity to state health headquarters and easy access (desirable district) compared to hard-to-reach districts (not at all desirable districts) that are sometimes far from state headquarters. As several districts were identified in each of the above category, three districts meeting the above criteria were selected from the different regions from the state for larger geographical representation.

### Data collection methods and sampling

#### Policy review

A brief policy review was carried out to understand the extant ACR-related policies, rules, and instruments. Under policy reviews, documents such as committee reports on ACR as well as the ACR form were looked at.

#### Interview with medical officers

This group consisted of classes I and II MOs who were the main subjects of the study. MOs working with only PHCs and CHCs and those from the block level were included in the study.

#### Sampling technique for medical officers

The study used purposive sampling at various stages while selecting the study respondents. This purposive selection approach focused on ensuring the representation of both male and female doctors: those with medical graduate degree and/or postgraduate medical degree and doctors from the block level and regular MOs that were GPSC confirmed from different geographical locations within the state. All this would not have been possible through random selection. Interviews with MOs were conducted till the time saturation in information was experienced. In total, 19 in-depth interviews were done with the MOs in Hindi.

### Data analysis

#### Document review analysis

Simple content analysis of the documents was done to understand the existing ACR-related rules and documents [24].

#### In-depth interviews and analysis

All interview recordings were transcribed verbatim and then translated into English text. Interviews were analyzed using thematic framework approach which is a matrix-based method to arrange and synthesize data [24]. The framework analysis approach was best suited to the scope of the current research as the aim of the research was to present themes identified in the data. To analyze the data, the study objectives, interview guide, and methodology adopted were regularly revisited. A framework approach was used to identify key words, themes, and sub-themes, and the transcripts of the 19 participants were coded and grouped according to the themes and sub-themes identified. A detailed analysis was performed using NVIVO 9 on the transcribed texts.

## Results

In this section, we first present the demographic profile of the respondents. Next, we present the overall ACR system in a descriptive way which gives a de jure (based on document review) as well as de facto (based on interviews) account of the ACR system such as how it works and what actually happens. Since the effectiveness of the appraisal system is assessed through features of the appraisal system presented in the conceptual framework of the study, the study results are categorized based on the themes identified as important for assessing effectiveness. These themes are as follows: the purpose of ACR; the source of ACR; the link of ACR to other HRM functions such as training, promotion, and salary administration; the feedback loop and quality; and finally the administrative effectiveness of the appraisal system. While presenting the findings under different themes, the perceptions and experiences of MOs about some of these key features of appraisals’ effectiveness as well the accuracy and fairness of current ACRs are also presented.“In the results sections” was changed to “In this section” as it appears more appropriate since the phrase is found in the “Results” section itself (in fact, it is the first sentence of the section). Kindly check if appropriate.Seems fine

### Demographic profile

The demographic profile of the study respondents is presented in Table 1. Although the study used purposive sampling to try and maintain gender balance by including lady medical officers (LMOs) (specific term used in India for female MOs), the overall availability of LMOs was very low and the present study could only include 3 LMOs out of 19 total respondents making it close to 16 %. As the state has good mix of ad hoc and bonded MOs, an almost equal representation of both the categories was reflected in the study.

**Table 1:** Distribution of MOs based on demographic and work profile

Gazetted Officer	District 1	District 2	District 3	Total
Class I	1	1	1	3
Class II	4	5	7	16
Gender				
Male	5	5	6	16
Female	0	1	2	3
Entered service through				
Bonded	3	4	4	11
Ad hoc	2	2	4	8
Place of work				
PHC	1	3	3	7
CHC	0	2	2	4
SDH/DH	3	1	0	4
BHO	1	0	3	4

#### The Annual Confidential Reports: a de jure account

The ACRs in India are governed by the All India Services Rules 1970. These rules provide that a confidential report or the ACR, assessing the performance of every member of the service, shall be written for each financial year [25]. The ACRs is the main method of annual periodic review of the performance of civil servants in India. In most states, the formats are uniform for all the employees regardless of the nature of functions. The ACR process is also meant to be used in training and human resource development, confirmation, etc. [26].

The ACR form, collected as part of a document review, and another committee report on ACR suggest that the ACR requires a general assessment by the “reporting officer” or the “appraiser” and a validation of these remarks by the “reviewing officer.” The appraisal is entirely confidential, with the exception that “adverse remarks,” if any, are required to be conveyed to the appraisee giving an opportunity to represent against such remarks. An ACR process in general requires (1) a self-appraisal from the appraisee and (2) an overall grade to be recorded (i.e., “outstanding”/“very good”/“average”/“below average”) by the reporting officer or “appraiser” and validated (or altered) by the reviewing officer. As the appraisal form has a section where the appraisee could record the training needs, with the comments of the reporting officer, the training needs section should be sent to the Training Division of the Department of Personnel and Training [25].

The main objective of ACR as explained under civil services in India is to provide basic and vital input for assessing the performance of an officer and for his/her advancement in his/her career, and it is a tool for human resource development so that an officer realizes his/her potential [15, 25].

#### The Annual Confidential Reports: a de facto account

The responses from MOs suggest that the ACR is an annual process where the reports are filled by the MOs by the end of the financial year and that the ACR form has a few sections. The first is a section on self-rating where MOs have the opportunity to self-rate themselves against certain performance indicators. Yet another section is where the appraiser puts the ratings about the appraisee categorized as follows: poor, average, good, and very good. The ACR forms also have a section where appraisees have an opportunity to write facilitating and inhibiting factors for their performance and a section where they can reflect training needs. The ACR forms once completed by the MO or the appraisee are sent to the reporting officers or the appraiser. The appraisers after writing their comments and ratings send it to reviewing officers for their final approval. Once the ACR reports are complete, these are sent to the Commissionerate and are in the overall custody of the chief personnel’s office (CPO).

### Source

The MOs responded that in the case of PHC and block-level MOs, the appraiser is the CDHO and the reviewing officer is a district development officer (DDO) whereas, in the case of CHC MOs, the appraiser is the superintendent and the reviewing officer is the regional deputy director (RDD).

#### Perception of medical officers about the source

Some of the MOs perceived that the appraiser as well as the reviewing officer (referred to as the “source” in the study framework) for PHC MOs may not be the right person to evaluate the performance of MOs as the DDO is not from the health field and does not understand the nature of work MOs are involved in.

"The DDO doesn’t even know where and what MO is doing, it’s the CDHO only who can tell him only then the DDO will know. Otherwise the DDO doesn’t know anything about our performance" (MO 10)

"The DDOs are not aware of anything. They are not having any knowledge about the work, they probably visit the PHC/CHC once or twice in a year. So the DDOs should not be the reviewing officers and this is the worst thing for the system" (MO 19)

### Purpose of Annual Confidential Report

According to document review, the main objective of ACR is to provide basic and vital input for assessing the performance of an officer and for his/her advancement in his/her career and it is a tool for human resource development so that an officer realizes his/her potential [15, 25].

However, the responses from MOs suggest that the main purpose of ACRs is to mainly make decisions relating to promotions and for higher grade. It was found that the negative remarks on ACR may affect promotions and higher grade.

"In case they [health department] need to promote us on performance basis or for salary increment purpose then ACR will be useful" (MO 10)

"ACR is useful for giving promotions-added responsibilities, higher pay scale, service related benefits and as a document in favor of MO that no adverse action has been taken against an MO during his/her service him and that he/she can continue services. And if the reviewing officers has put a negative remark about an MO then in future an MO faces problems" (MO 15)

"Negative ACR can work against the person not getting higher grade" (MO 1)

### Feedback mechanisms

Responses from MOs suggest that there is no feedback mechanism in place to discuss the performance assessed through ACRs. The study found that in the whole process of ACR there is no formal way of sharing the ratings, comments, and feedback by the appraiser with the appraisee. Only in the case when remarks are adverse or negative and the overall performance is judged to be poor, the appraiser is required to share and discuss it with the appraisee. However, most of the MOs said that negative remarks are generally not given by the appraisers.

"In case a reporting officer writes something positive then you [MO] would never know that. I have never known what my ACR report says" (MO 16)

"If the CDHO has any negative remarks on your [MOs] performance then he has to inform the concerned doctor. CDHO cannot write negative remarks without informing the concerned MO. However if it’s a positive remark, CDHO would never informs. If CDHO has a negative remark, then MO gets a chance to explain his position" (MO 1)

"No we don’t get to know about what a reporting officer writes on our ACR. But if it is bad then we do get to know as there is a policy. If my CR is bad then I’ll get to know on paper that my CR is bad, and if I have to say anything in that regard" (MO 17)

"If it’s [ACR] good, we don’t come to know. But if the remarks are bad or negative by CDHO then it’s the responsibility of the reviewing officer to notify the MO that these are the comments given my reporting officer. In this process I am given the chance to give justification and if the reviewing officer finds the justification good enough then he has the power to change my A CR" (MO 2)

"Generally they don’t put anything adverse. You have to be a very poor doctor or you have to have antagonized your superior so much that they could spoil it" (MO 16)

### Link of performance appraisal system with other human resource management functions: training, promotion, and salary administration

Most of the MOs perceived that ACRs have no link to identify training needs or to assess performance and perceived that it is mainly used for promotion-related decisions.

"The ACR is not at all used for identifying training needs. For training seniority is considered" (MO 1)

#### Perceptions about use of the Annual Confidential Report system

According to a few MOs, ACRs were considered to be used in maintaining discipline and in assessing performance and to show power and authority by the appraiser or to take disciplinary actions against the appraisee.

"No one looks at the ACR and they close the ACRs as soon as they reach commissioner’s office. I have never sat with my reporting officer to discuss it. I think ACR is just a formality" (MO 1)

"There is no use of ACR in the government. It is used only for penalizing. It is a totally useless system" (MO 19)

"The ACR system is useful. With ACR the reporting officer can command authority with it and it is useful to have some control over MOs. If there is no CR, who would listen to senior reporting officer. It brings some discipline. MOs will have a fear that if we do not listen to our boss then the senior may spoil our CR. It is also useful to assess performance of a MO in the year. But I feel that if someone does well then he should be given some recognition and encouragement for his work" (MO 12)

### Administrative effectiveness and its implications

Although the ACRs were reported to be used for administrative decisions such as promotions and higher pay scale, several MOs reported that their completed or filled ACRs for the past years were missing from the repository and that they got to know about it only when their promotions or salary increments were due. Further, the MOs reported that they had to go through inconvenience to get their past missing ACRs completed again.

"The ACRs generally do not get stuck, but the persons whose ACR gets stuck only realizes its importance. Only when the list for promotion etc is prepared, the candidate [MO] comes to know that his ACR is missing. If we know in advance about such situations, then we can make photocopies of the ACRs. And if CRs are missing, the person may not be eligible for the higher grade" (MO 1)

"The people at headquarters told me that my ACRs were missing for which particular years. Although I told them that had filled and completed ACRs for all the years and sent it at the Zila [district] level. On enquiring with Zila I found that my CRs were not with them and that I needed to fill these again in order to get higher salary grade which was due for last six years. I had to get my missing ACRs filled and signed again by the DDO who was in that district at that time for which my CR was missing. I had to run around very much to get it completed". (MO 11)

"I received a letter that my ACR is pending and I was asked to fill it up gain and send it. According to the CPO officer, my CRs from 1995 to 2002, 2006 - 08 were pending however I got the letter for the missing ACRS only two months back [around April 2014]. In the last 18 years of my service, this is the first time that I received such a letter from the Govt. that my CRs are pending. I know that I had completed and sent all my ACRs in time. The government should see, that I have completed 7 years of service and that I should get higher grade and they should inform in time that ACRs are missing but they don’t tell. Not only I but other MOs have also faced such problems. Problem is more with the Zila panchayat" (MO 13)

The general perception about the actual process of those whose ACRs were missing was negative, and they were of the opinion that such ACRs were lost at the district level.

"I am telling you, most likely 90 % of the ACRs are lost here, at the District Panchayat. They are not sending the CRs ahead, the CRs are being suppressed, they are being god knows what…it doesn’t leave the District Panchayat. Some of them could be intentional, some of them could be because of the inefficient system at clerk level. The clerks who are handling it are also corrupt. Many of them are corrupt. Like suppose you are the clerk there who is handling this now some doctors may come and pay you so your CRs may go ahead. That sort of a thing" (MO 16)

"This happens at administrative level, clerk level or administrative officer level. I don’t think they do it on purpose. They have to keep a record of so many doctors, so it is possible that 1 or 2 get misplaced in the process or is not sent. The system to fill the CR is also a little complicated. The problem probably arises when he is transferred in between or the DDO changes, maybe it doesn’t really happen on purpose". (MO 17)

### Accuracy and fairness

Several MOs felt that the assessment of performance and the final ratings on ACR are contingent upon the personal relationship a MO or an appraisee has with the reporting officer or the appraiser which reflects that the appraisals may not be accurate and may not achieve the desired objective of assessing performance accurately. Further, several MOs perceived the appraisal system to be just a formality where performance is not accurately reflected.

"If you have good relations with your superiors then you will get good remarks. If you speak well with your CDHO, you will get good remarks. And if you do not agree with him on anything, then ACR may have some negative or not so good remarks" (MO 10)

"100 %, I feel that relationship of an MO with reporting officer affects the ACR. This relationship with the higher authority, determines if you get positive remarks or negative. It happens in all the departments of the government" (MO 15)

## Discussion

The study aimed at understanding the current ACR systems and their effectiveness. The five main features of an effective appraisal system studied in the current research (purpose, source, feedback quality, link of ACR to other HRM functions, and administrative effectiveness) indicate that the overall appraisal system is ineffective and may have an influence over appraisees’ perceived accuracy about the appraisal system.

As far as the first characteristic of an effective appraisal system, i.e., purpose, is concerned, it can be concluded that the current ACR system is not effective in meeting the actual intended objectives of the ACR in relation to employee development.

The next appraisal characteristic, which is source of appraisal, was also perceived to be not very favorable by the MOs indicating ineffectiveness. The study respondents in several cases indicated that the reviewing officer may not be the appropriate person to be evaluating the final performance of the appraisee. While validation of the appraisal report by the reviewing officer may be used or in place as a control mechanism to prevent patronage or bias as a result of personal relationship between appraiser and appraisee, the overall systems of validation by the reviewing officer were not looked at very favorably be several MOs. This puts a question on whether the health department should think about having reviewing officers who are from the health field and those who understand the nature of work MOs or appraisees are doing.

As far as feedback quality is concerned, it can be concluded that the current ACR system has communication gaps and is influenced by personal relationships indicating personal biasness and there is a lack of employees’ participation. This not only makes the ACR system ineffective but such a system does not result in employee learning and development. Similar findings have been reported from other MLICs about the ACR [16]. Such a system also fails to achieve the intended objective of employee development under the ACR of civil services [15, 25] and of a good appraisal system that is aimed at contributing to the development of people by identifying their strengths and weaknesses and providing feedback on the same and also providing a support system where individuals can be provided training and counselling based on issues identified in the appraisal reports. Research in performance appraisal has demonstrated that performance appraisal characteristics (such as appraisal purpose and source) can elicit positive employee reactions to performance appraisal and which, in turn, can motivate employees to improve their performance [27]. As far as the administrative effectiveness is concerned, the study found the ACR systems to be ineffective as several appraisees reported that their ACRs were missing from government records.

ACR in the current study was found to be subjective and unilateral in character, as has been described in another document [26]. Although the quotes from the study do not directly reflect the perceived accuracy, the quotes suggest that the ACR system fails to assess performance correctly and that such ratings are based on personal relationship rather than actual performance. Perceived accuracy may further influence the main appraisal outcomes: satisfaction and motivation.

The ACR system operates in isolation with no link to training and a weak link with salary administration and promotion where all MOs (irrespective of performance, except those who get poor rating) may be treated at par. This also indicates that performance is not clearly linked to rewards having implications for MO motivation and a question as to why MOs should strive hard to perform well. The ACR is only linked to the salary administration and promotion of appraisees subject to satisfactory ACR reports. However, the link of the ACR system with salary administration and promotion is not linear to performance as the promotion-related decisions are based on seniority.

As far as accuracy and fairness of the ACR system is concerned, it can be said that the study respondents considered the ACR system to be a formality or a ritual and a system where MOs hardly get to know about their performance, especially good performance. Therefore, it can be concluded that the current ACR system fails to achieve the intended objective of contributing towards employee development. Further, the feedback loop, one of the most important features of an effective appraisal system which is linked to appraisal outcomes such as improved performance [20, 28, 29], was found missing. The study results indicate that the ACR system majorly focuses on discussing the negative ratings with no scope for reinforcement of positive aspects having implications for MO motivation, especially with regard to appreciation and recognition [30].

## Conclusions

In light of the ineffectiveness of the current ACR system as identified in the study, several recommendations are suggested. There is no great need in designing a new format for ACR as the current formant if used effectively can contribute to achieving the appraisal objectives of employee development, assessing performance, and career development. However, there is a great need for having systems in place or designing systems that can ensure that the ineffectiveness of the current ACR system in all five domains can be addressed. The need for designing effective performance appraisal systems that can motivate employees in order to improve employee performance has been highlighted [27].

The findings from the study indicate that the ACR system is not effective in all the five domains. Hence, we suggest the following ways in which the effectiveness of the current ACR system may be improved. Firstly, it is recommended that the reporting officers or appraisers in order to make the ACR system more effective can have more frequent and timely communication with the MOs. This is particularly important as discussions and feedback on performance between the evaluator and employee being evaluated in the ACR systems in India have found to be infrequent and typically discussions about performance only take place in the case of adverse remarks [26]. Such a communication can particularly focus on giving constructive feedback to the MOs for their overall development so that MOs can work on their strengths and improve over their weaknesses. This recommendation is particularly important as HRM literature suggests that performance appraisal systems must focus on the development of employees and giving constructive feedback is one of the ways to ensure employee development [1]. Further, the communication should be two-way where appraisees get to know the comments, ratings, and feedback given by the appraisers, both positive and negative. Although the current ACR form has a section to identify the training needs of MOs, the system does not systematically identify the training needs of the MOs. Therefore, it is suggested that the section on identifying training needs to MOs should be effectively used to design training programs based on the needs identified in such ACR forms as this would help in achieving the intended objective of the ACR system to provide employees advancement of their career and for human resource development [26].

Addressing the five characteristics of an effective appraisal system can lead to improved perceived fairness MOs have from the current ACR system which may further influence satisfaction and motivation positively. Improved motivation and satisfaction with the appraisal system can influence two important HRH-related outcomes, i.e., performance and retention.
